# Diversity of spined loaches from Asia Minor in a phylogenetic context (Teleostei: Cobitidae)

**DOI:** 10.1371/journal.pone.0205678

**Published:** 2018-10-11

**Authors:** Anabel Perdices, Cevher S. Ozeren, Füsun Erkakan, Jörg Freyhof

**Affiliations:** 1 Department of Biodiversity and Evolutionary Biology, Museo Nacional de Ciencias Naturales, CSIC, Madrid, Spain; 2 Department of Biology, Faculty of Science, University of Ankara, Ankara, Turkey; 3 Department of Biology, Faculty of Science, Hacettepe University, Beytepe Campus, Ankara, Turkey; 4 Leibniz-Institute of Freshwater Ecology and Inland Fisheries (IGB), Berlin, Germany; Southwest University, CHINA

## Abstract

Accurate determination of species diversity in areas of high endemicity, particularly those lacking comprehensive systematic knowledge, represents a challenge for both taxonomists and conservationists. This need is particularly evident in areas greatly affected by anthropogenic disturbances such as the Eastern Mediterranean and its freshwater environments. To improve our knowledge of Eastern Mediterranean freshwater fishes, we phylogenetically studied Western Palearctic *Cobitis* species, focusing on those found in Turkey. Overall, our results provide a robust framework to assess the number of species of *Cobitis*. Phylogenetic reconstructions based on mitochondrial (cyt b) and nuclear (RAG1) sequences show seven major clades (Clades 1–7) grouping all Western Palearctic *Cobitis* species, except *C*. *melanoleuca*. In general, each major clade comprises *Cobitis* species that inhabit geographically close areas and have similar secondary sexual characters. Multiple divergent lineages were identified in our analyses, some of which were highly divergent such as the ones inhabiting Turkish freshwaters. Moreover, in some analyses, several of the identified lineages were incongruent with *a priori* defined species. Furthermore, our analyses identified eight potentially new candidate species, six that had been suggested in previous studies and two that are reported here for the first time. Our results reveal Turkey as the area with the greatest diversity of spined loaches in the Mediterranean.

## Introduction

Accurate assessment of the number of species and correct species identification are fundamental to systematic biology. Correct species identification and knowledge of endemism patterns are particularly important for biodiversity studies, endangered species lists and the prioritization of regions for conservation [1,2]. Precise species recognition is especially challenging in groups exhibiting very low morphological variation but is crucial for adequate conservation strategies, particularly of geographical regions rich in endemic species under strong anthropogenic pressure, such as those in the Eastern Mediterranean [3].

Renewed interest in assessing biodiversity levels has been driven by the implementation of molecular COI DNA barcoding [4] and the emergence of new analytical methods for species delimitation [5–7]. Great effort has been invested into building a global COI DNA reference library (as well as one with the less universal cytochrome b gene) which has significantly improved our knowledge of biodiversity in different regions, resulting in the recognition of so-called “well-known areas”, which include Europe and adjacent countries [8]. However, COI barcoding has been criticized for its use of genetic distances to assess phylogenetic relationships [9] and its inability to accurately delimit species, particularly in young evolutionary lineages [10] and introgressed species [11]. In barcode studies, mitochondrial genes are typically used as provisional identifiers at the species level [4], and only in combination with analyses of nuclear markers have they then been able to reveal controversial phylogenetic relationships. Therefore, evidence from multiple independent lines of enquiry are generally needed to shed light on species boundaries as no clear or universal signals are offered by single markers [12].

In this study, we examine the spined loaches of the genus *Cobitis*, a group of small benthic freshwater fishes found on sandy river bottoms and in Palearctic lakes [13]. Kottelat [14] noted the complex systematic situation of this genus and the provisional character of its taxonomy: at that time, he recognized 65 *Cobitis* species, 42 of which occur in the Western Palearctic. European *Cobitis* species have been reviewed in several studies [13], [15], [16]; however, the Western Asian species remain poorly studied. Only a couple of local revisions [17,18] and individual species descriptions [19–24] of *Cobitis* species in this region have been published thus far.

The Western Palearctic, which includes Mediterranean drainages in Asia, circum-Black and Caspian seas, Mesopotamia and Iran, among other regions, is recognized for its high level of endemism of freshwater fishes [25,26]. A large number of *Cobitis* species have been described as being endemic to this area; however, a complete overview of *Cobitis* species of the Western Palearctic has not been published to date. One of the most comprehensive studies of freshwater fishes in this area is the barcode study by [10], which included Mediterranean species but excluded all non-Mediterranean European species, including those from the Black and Caspian Sea basins and the Persian Gulf basin. On the basis of the genetic and morphological distinctiveness found in their study, the authors proposed the existence of at least 64 unrecognized candidate species of Mediterranean freshwater fishes, including nine *Cobitis* species [10]. Two of these *Cobitis* species were recently described as *Cobitis sipahilerae* and *Cobitis doraderimi* from Anatolian waters [21].

Here, we assess species diversity of *Cobitis* in the Western Palearctic, particularly of the Anatolian representatives, by analysing mitochondrial cytochrome b (cyt b) and nuclear Recombination-activating gene 1 (RAG1) genes. We present a comprehensive phylogeny of Western Palearctic *Cobitis* species to test mtDNA-based phylogenies and the number of species. Overall, our data provide a robust phylogenetic framework to evaluate *Cobitis* species diversity. We also characterize patterns of genetic variation in these species to identify endemic areas and to determine the relationships of the different phylogenetic groups of *Cobitis* [27–29].

## Material and methods

This investigation was conducted in accordance with ethical standards and Spanish legislation. Approval from the Ethics committee was not necessary since wild fauna was excluded in LAW 32/200 of 7th November 2007 (BOE 8/11/2007) which regulates the experimental use of animals in Spain. No endangered species were used in this study. Collection permits were obtained for electrofishing with the authorization to collect fin tissue and/or sacrifice few specimens. The sampling was conducted by electrofishing, and the specimens were sacrificed with an overdose of the anaesthetic MS-222 (tricaine methanesulfonate) and/or preserved in 95% ethanol in the field. Permission for sampling in Turkish waters was issued by the Ministry of Food of the Republic of Turkey.

### Taxon sampling and data analyses

**Fig 1:**
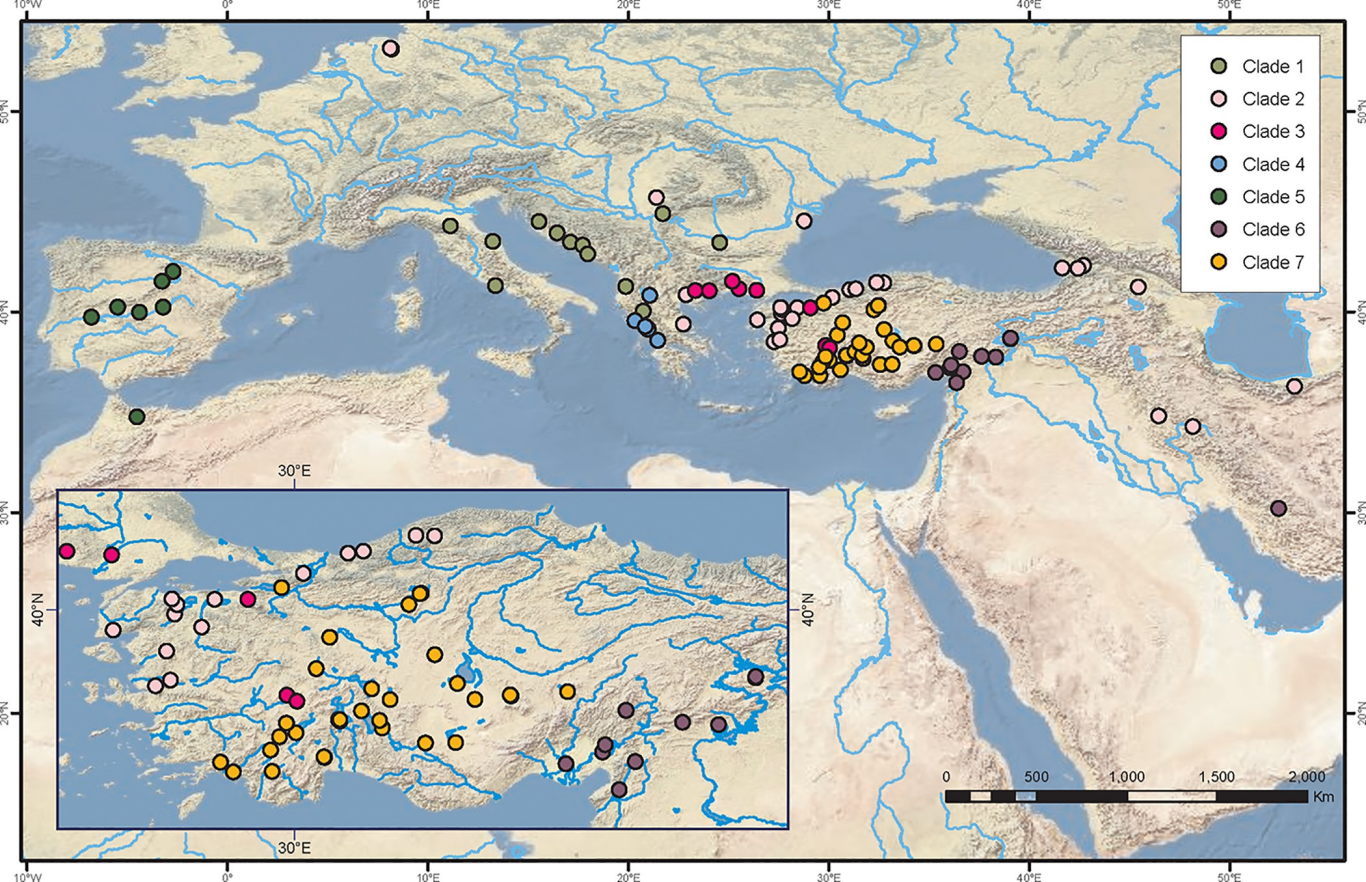
Map showing sampling localities. The distribution of major Clades 1–7 of Western Palearctic *Cobitis* recovered in the molecular phylogenies are also shown. Major clades are indicated with different colours, which are maintained in Figs 2–4.

We identified species according to their morphology and geographical distribution following previous studies [13], [15], [17], [21]. We used external secondary sexual characters for *Cobitis* identification, specifically relying on a character known as the *lamina circularis* or scale of Canestrini, a thickening of the pectoral rays on *Cobitis* males. The absence, presence or duplication of this character provided the basis for identification. In the time since [14] published a global checklist of loaches, four new species have been described for the area. Therefore, in this study, we recognized a total of 46 valid species for the Western Palearctic [19], [21], [23] and molecularly analysed 41 of these species (Fig 1, S1 and S2 Tables). For phylogenetic analyses, we used *C*. *choii* as the primary outgroup [29] and *Sabanejewia aurata*, *Oxynoemacheilus hanae*, *O*. *gyndes*, *Pangio pangia* and *Kottelatlimia pristes* as more distant outgroups. If possible, specimens were sampled from multiple localities across the known range of each species. In order to place Eastern Mediterranean *Cobitis* species into a broader phylogenetic context, we first analysed the 41 selected species with other spined loaches of the *Cobitis* sensu lato group that are included in the Northern Clade defined by [29] (S1 Fig).

The full-length mtDNA cyt b (1140 bp) and nuclear RAG1 (877 bp) genes were analysed as described by [30]. These genes were selected for phylogenetic reconstructions as previous analyses revealed both genes to be informative at different phylogenetic depths [28]. We combined data obtained from previous studies [27–29] with new data obtained in this study from Western Asia, particularly Turkey. When possible, we isolated both genes from the same specimen; otherwise, any available sequences were used for the analyses. A total of 211 new sequences were obtained and deposited into GenBank (for accession numbers, see S1 and S2 Tables).

Total DNA was extracted from fins using the ChargeSwitch gDNA Micro Tissue Kit (Invitrogen, Carlsbad, CA, USA). The conditions and primers used for PCR amplifications of cyt b and RAG1 were as previously described by [30,31]. Purified PCR products were sequenced directly using the primers used for amplification.

### Phylogenetic analyses

We used the complete cyt b (N = 199) and complete RAG1 (N = 158) datasets for gene tree reconstructions. Phylogenetic trees were inferred for the complete datasets using Maximum Likelihood (ML) and Bayesian inference (BI) approaches. Robustness of inferred trees was assessed by bootstrapping (1000 replicates) in ML analyses and posterior probability (pp) values in BI analyses. The ML method was implemented in RAxML [32] through its graphical interface RAxMLGUI 1.3 [33], using the GTR+I+G model of evolution with 1000 bootstraps (BT). All trees were used to construct a 50% consensus tree in PAUP* v. 4.0a146 [34] for each dataset. The BI method was implemented in MrBayes v.3.1.2, partitioning the dataset by codon position for cyt b and under a GTR+I+G substitution model (nst = 6 and pinvGamma). We ran four simultaneous Monte Carlo Markov Chains (MCMC) for 2 million generations, sampling every 1000 generations, chain temperature 0.2. Log-likelihood stability was attained after 10,000 generations, and we excluded the first 100 trees as burn-in. The remaining trees were used to compute a 50% majority rule consensus tree in PAUP*. We performed a ML analysis in RAxMLGUI using a concatenated dataset of the two markers (N = 154), and the GTR+I+G model of evolution with 1000 bootstraps (BT). A BI analysis was also performed on the combined dataset, independently by gene, using the parameters described above. For the broader phylogenetic analysis of *Cobitis* species, we performed a ML analysis in RAxMLGUI (GTR+I+G,1000 BT) using an extended (N = 215) concatenated dataset (cyt b and RAG1) that contained species included in the *Cobitis* sensu lato group [29].

All taxa have the same number of codons for cyt b and RAG1 with no stop codons when translated to amino acid sequences. No gaps or ambiguous alignments were found, and all positions were used in the analyses. Sequences were checked and aligned with Sequencher ver. 4.8 (Gene Codes Corp., Ann Arbor, MI, USA).

### Species delimitation and K2P sequence divergence

To identify potential species boundaries within our dataset, we used the Poisson tree processes (PTP) model proposed by [35], [36], and the maximum likelihood (ML) phylogeny generated with the complete cyt b dataset. We used two versions of the PTP model: bPTP, which adds Bayesian support (pp) values to branches that delimited species in the input tree, and the refined multi-rate mPTP. Both bPTP and mPTP are single locus species delimitation methods that use non-ultrametric phylogenies in which the number of substitutions are directly reflected by branch lengths. A key assumption is that the number of substitutions between species is significantly higher than the number within species. The RAxML gene tree was then used as the input for the PTP analyses as implemented on the PTP server, with 100,000 MCMC generations, a thinning of 100 and a burn-in of 0.1.

We used the complete cyt b dataset comprised of 199 spined loach sequences to calculate Kimura 2 parameter (K2P) distances [37] for direct comparison with the barcode (COI) reference database of Mediterranean freshwater fishes that includes multiple *Cobitis* species [10] [we excluded one *C*. *bilineata* specimen that exhibited a highly discordant COI sequence (GenBank ID = KJ553116)]. Mean K2P distances (range and standard deviation) using the “pairwise deletion option” were calculated in Sequencer 6.1 (written by B. Kessing). We estimated K2P sequence divergence among the major lineages recovered in the RAxML gene tree and between species delimited with PTP methods. These analyses will allow us to compile values for species identified on the basis of morphology and for molecular lineages delimited by analyses of cyt b sequences that will then be used in subsequent studies to confirm potentially new *Cobitis* species based on COI sequences [10].

## Results

### Phylogenetic analyses

**Fig 2:**
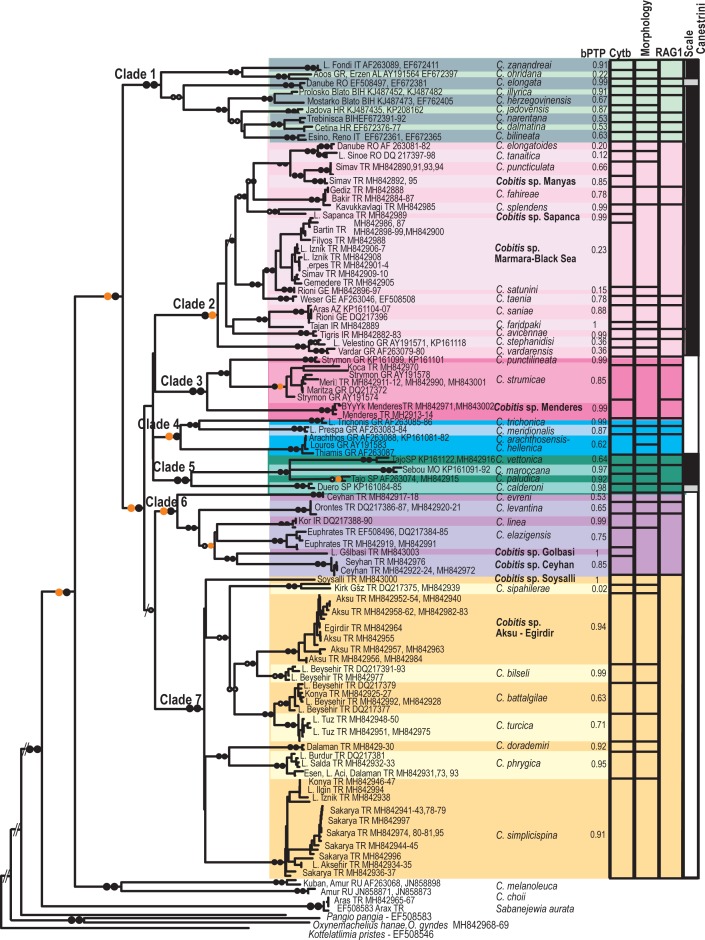
Phylogenetic tree for Western Palearctic *Cobitis* based on the complete cyt b dataset (N = 199). Symbols on branches correspond to bootstrap values (BT) for RAxML and Bayesian posterior probability support (pp), respectively: Black dots >95 BT/pp, orange dots 85–95 BT/pp, grey dots <85–50 BT/pp, / No support. To the right of the phylogeny, the column labelled bPTP represents Bayesian posterior probabilities (pp) for hypothesized species obtained by bPTP analyses. The coloured bars to the right represent hypothesized species and species groups based on the BT/pp values of the complete cyt b and RAG1 datasets and identified species according to their morphology with the disclosed bar Scale Canestrini indicating the number (0–2) of *lamina circularis* or scale of Canestrini exhibited by *Cobitis* males: 0 grey, 1 black, 2 white. The seven major clades (Clades 1–7) obtained in the mtDNA analysis are represented by the different colours following those shown in Fig 1. Individual species within clades are identified using darker to lighter shades of the corresponding clade colour.

**Fig 3:**
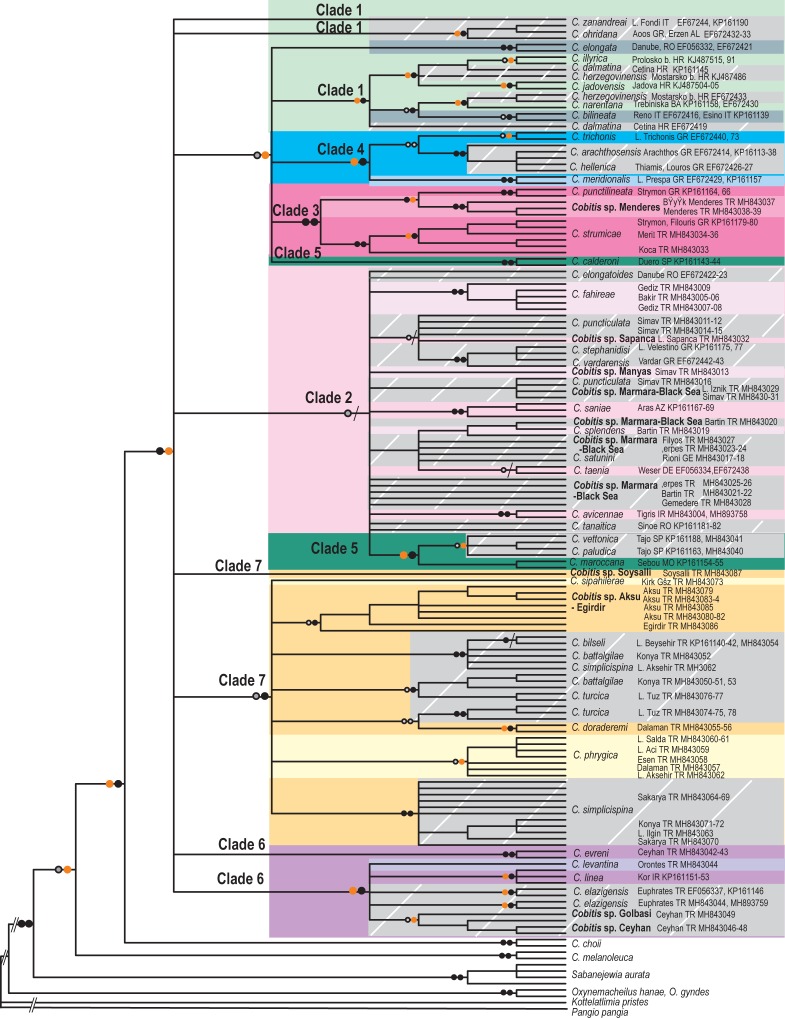
Phylogenetic tree for for Western Palearctic *Cobitis* based on the RAG1 dataset (N = 158). Symbols on branches correspond to bootstrap values (BT) for RAxML and Bayesian posterior probability support (pp), respectively: Black dots >95 BT/pp, orange dots 85–95 BT/pp, grey dots <85–50 BT/pp, / No support. The seven major clades (Clades 1–7) obtained in the mtDNA analysis are indicated in the RAG1 phylogeny with similar colour as shown in Fig 1. Species of each major clade are identified with darker to lighter shades of the corresponding clade colour. Species not recovered as monophyletic are indicated with grey boxes with bars.

**Fig 4:**
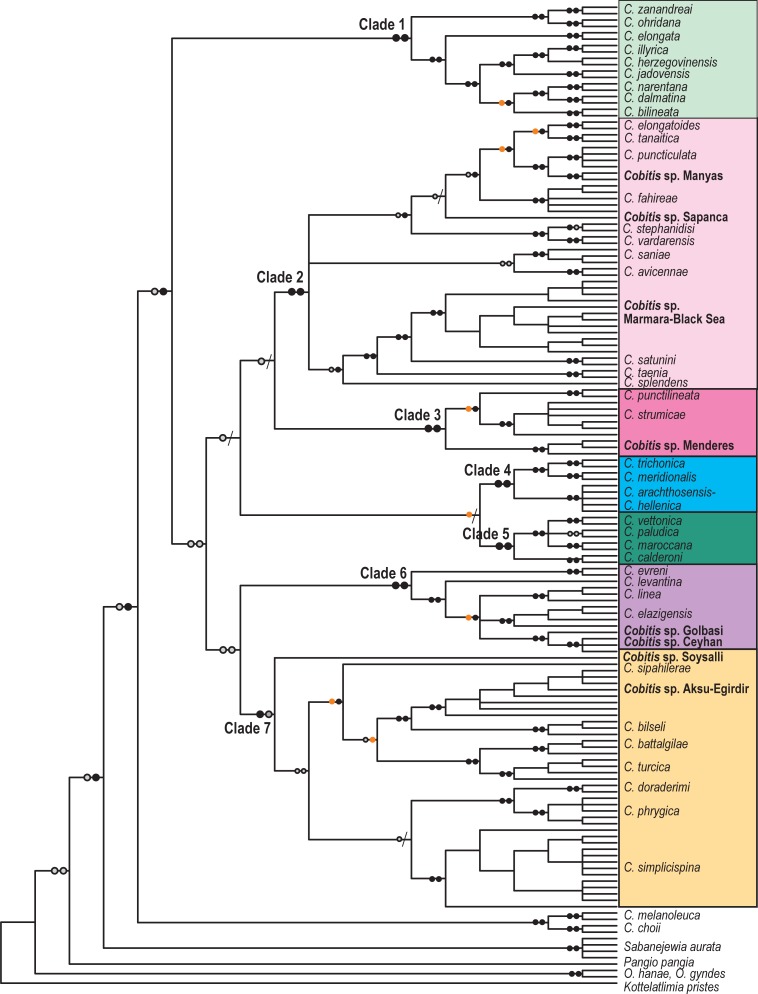
Phylogenetic tree of the combined dataset (cyt b + RAG1) (N = 154) for Western Palearctic *Cobitis*. Symbols on branches correspond to bootstrap values (BT) for RAxML and Bayesian support (pp). Black dots >95 BT/pp, orange dots 85–95 BT/pp, grey dots <85–50 BT/pp, / No support. The seven major clades (Clades 1–7) obtained in the mtDNA and combined analyses are represented by the different colours as shown in Fig 1.

In the extended phylogenetic analysis, *Cobitis* species described for the Western Palearctic were included in Subgroup II of the *Cobitis* sensu lato group recovered by [29]. This subgroup contains species that are widespread across Europe Asia Minor, the Black Sea and the Caucasus, with some lineages related to species restricted to East Asia (S1 Fig). All analyses resolved a Western Palearctic clade that included 40 previously described species of *Cobitis* from this region; the clade is further organized into seven major phylogenetic clades (Clades 1–7) with unresolved basal relationships (Figs 2–4, S1 Fig). *Cobitis melanoleuca* does not belong to the Western Palearctic clade but rather is more closely related to East Asian species. All seven clades were well supported in phylogenies derived from the complete cyt b and combined datasets (>87BT, >0.94pp). Only partial groups were supported in the more conserved nDNA phylogeny based on the complete RAG1 dataset (Table 1 and Fig 3).

**Table 1:** List of *Cobitis* species analysed. Support values (BT/pp) are also provided for the different analyses: bPTP, ML and BI using the complete cyt b, complete RAG1 or combined datasets. NO refers to species not supported in the bPTP analysis or COI barcode study [10]. COI refers to values found by [10]; NO/NO refers to species not supported in ML/BI analyses, respectively.–indicate species not included in the analyses; 1 indiv = 1 specimen analysed. *Lamina circularis* (scale of Canestrini), a thickening of the pectoral rays on *Cobitis* males, refers to its absence (0), presence (1), or duplication (2).

MtDNA Clade	Morphological identification	bPTP (N = 199)	Complete cyt b ML/BI (N = 199)	COI (N = 127)	Complete RAG1 ML/BI (N = 158)	Combined cytb & RAG1 ML/BI (N = 154)	*Lamina circularis*
Clade 1	*C*. *bilineata*	0.63	100/1	99	81/1	100/1	1
Clade 1	*C*. *dalmatina*	0.53	100/1	97	NO/NO	99/1	1
Clade 1	*C*. *elongata*	0.99	100/1	_	96/1	100/1	0
Clade 1	*C*. *herzegovinensis*	0.67	100/1	_	NO/NO	100/1	1
Clade 1	*C*. *illyrica*	0.91	100/1	100	64/0.99	99/1	1
Clade 1	*C*. *jadovensis*	0.87	100/1	100	89/1	100/1	1
Clade 1	*C*. *narentana*	0.53	100/1	92	NO/NO	100/1	1
Clade 1	*C*. *ohridana*	0.22	99/1	99	NO/NO	100/1	1
Clade 1	*C*. *zanandreai*	0.91	100/1	100	NO/NO	100/1	1
Clade 2	*C*. *avicennae*	0.99	100/1	_	100/1	100/1	1
Clade 2	*C*. *elongatoides*	0.20	100/1	_	NO/NO	100/1	1
Clade 2	*C*. *fahireae*	0.78	100/1	100	99/1	100/1	1
Clade 2	*C*. *faridpaki*	1	1 indiv	_	_	_	1
Clade 2	*C*. *puncticulata*	0.66	100/1	100	NO/NO	100/1	1
Clade 2	*C*. *saniae*	0.88	100/1	_	100/1	100/1	1
Clade 2	*C*. *satunini*	0.15	100/1	_	NO/NO	100/1	1
Clade 2	*C*. *splendens*	0.99	1 indiv	100	1 indiv	1 indiv	1
Clade 2	*C*. *stephanidisi*	0.36	100/0.71	_	NO/NO	100/0.64	1
*Clade 2*	*C*. *taenia*	0.78	100/1	_	63/NO	100/1	1
Clade 2	*C*. *tanaitica*	0.12	100/0.99	_	NO/NO	100/1	1
Clade 2	*C*. *vardarensis*	0.36	100/1	100	NO/NO	100/1	1
Clade 2	*Cobitis* sp. Sapanca	0.99	1 indiv	100	1 indiv	1 indiv	1
Clade 2	*Cobitis* sp. Manyas	0.85	100/1	_	NO/NO	100/1	1
Clade 2	*Cobitis* sp. Marmara-Black Sea	0.23	95/1	_	NO/NO	97/1	1
Clade 3	*C*. *punctilineata*	0.99	100/1	100	100/1	100/1	2
Clade 3	*C*. *strumicae*	0.85	91/1	100	99/1	100/1	2
Clade 3	Cobitis *sp*. Menderes	0.99	100/1	100	99/1	100/1	2
Clade 4	*C*. *arachthosensis*	NO	NO/NO	NO	NO/NO	NO/NO	2
Clade 4	*C*. *hellenica*	NO	NO/NO	NO	NO/NO	NO/NO	2
Clade 4	*C*. *meridionalis*	0.87	100/1	100	98/1	100/1	2
Clade 4	*C*. *trichonica*	0.99	100/1	100	92/0.53	100/1	2
Clade 5	*C*. *calderoni*	0.98	100/1	100	98/1	100/1	0
Clade 5	*C*. *maroccana*	0.97	100/1	100	72/0.99	100/1	1
Clade 5	*C*. *paludica*	0.92	71/0.84	NO	NO/NO	76/0.83	1
Clade 5	*C*. *vettonica*	0.64	100/1	NO	NO/NO	100/1	1
Clade 6	*C*. *elazigensis*	0.75	100/1	_	NO/NO	100/1	2
Clade 6	*C*. *evreni*	0.53	100/1	100	98/1	100/1	2
Clade 6	*C*. *levantina*	0.65	100/1	100	1 indiv	1 indiv	2
Clade 6	*C*. *linea*	0.99	100/1	_	89/1	100/1	2
Clade 6	*Cobitis* sp. Ceyhan	0.85	100/1	100	55/0.89	98/1	2
Clade 6	*Cobitis* sp. Golbasi	1	1 indiv	100	1 indiv	1 indiv	2
Clade 7	*C*. *battalgilae*	0.63	89/1	100	NO/NO	100/1	2
Clade 7	*C*. *bilseli*	0.99	92/0.98	96	NO/NO	100/1	2
Clade 7	*C*. *doraderemi*	0.92	100/1	100	93/1	100/1	2
Clade 7	*C*. *phrygica*	0.95	100/1	99	NO/NO	100/1	2
Clade 7	*C*. *simplicispina*	0.91	99/1	_	NO/NO	100/1	2
Clade 7	*C*. *turcica*	0.71	100/1	99	NO/NO	100/1	2
Clade 7	*Cobitis* sp. Aksu-Eğirdir	0.94	100/1	_	76/1	100/1	2
Clade 7	*C*. *sipahilerae*	0.02	100/1	100	1 indiv	1 indiv	2
Clade 7	*Cobitis* sp. Soysalli	1	1 indiv	1 indiv	1 indiv	1 indiv	2

Each clade was comprised of geographically close *Cobitis* species that display similar secondary sexual characters, namely in the number of *lamina circularis* (Fig 2), congruent with previous studies that had less geographic coverage of species [27], [29], [30], [38].

In the mtDNA and combined analyses, nine *Cobitis* species from the Adriatic and upper Danube drainages (*C*. *bilineata*, *C*. *dalmatina*, *C*. *elongata*, *C*. *herzegovinensis*, *C*. *illyrica*, *C*. *jadovensis*, *C*. *narentana*, *C*. *ohridana* and *C*. *zanandreai*) comprised Clade 1 with strong support (>98BT, 1pp) (Figs 2 and 4). Species from Central and Eastern Europe and the Asian Black Sea basin resolved as a monophyletic group identified as Clade 2 (>97BT, 0.94pp). This group included at least twelve previously described species (*C*. *avicennae*, *C*. *elongatoides*, *C*. *fahireae*, *C*. *faridpaki*, *C*. *taenia*, *C*. *tanaitica*, *C*. *puncticulata*, *C*. *saniae*, *C*. *satunini*, *C*. *splendens*, *C*. *stephanidisi* and *C*. *vardarensis)* and three potentially undescribed species (*Cobitis* sp. Sapanca, *Cobitis* sp. Manyas, *Cobitis*. sp. Marmara-Black Sea). Clade 3 (100BT, 1pp) consisted of two described and one undescribed species of *Cobitis* from eastern Greece and western Turkey (*C*. *strumicae*, *C*. *punctilineata* and *Cobitis* sp. Menderes). Clade 4 (>87BT, 0.99pp) was comprised of four species inhabiting small areas of western Greece: two of the species (*C*. *meridionalis* and *C*. *trichonica*) were recovered as reciprocally monophyletic; however, the other two (*C*. *arachthosensis* and *C*. *hellenica*) did not resolved as distinct groups. Clade 5 (>98BT, 0.99pp) included the species from the Iberian Peninsula and Morocco (*C*. *calderoni*, *C*. *maroccana*, *C*. *paludica* and *C*. *vettonica*), which resolved as reciprocally monophyletic. Clade 6 (>86BT, 1pp) was composed of four described and two potentially undescribed species from the easternmost areas of the Mediterranean, Iran and the upper Euphrates drainage (*C*. *elazigensis*, *C*. *evreni*, *C*. *levantina*, *C*. *linea*, *Cobitis* sp. Ceyhan and *Cobitis* sp. Gölbasi). Finally, Clade 7 (98BT, 1pp) included seven *Cobitis* species and two potentially undescribed species inhabiting Central and Southwest Anatolia (*C*. *battalgilae*, *C*. *bilseli*, *C*. *dorademiri*, *C*. *phrygica*, *C*. *simplicispina*, *C*. *turcica*, *C*. *sipahilera*e, *Cobitis* sp. Aksu-Eğirdir and *Cobitis* sp. Soysallı).

#### Nuclear gene analyses

The consensus nuclear phylogeny showed moderate to high support (97BT, 0.67pp) for a Western Palearctic clade but was generally less informative for major groups (Fig 3). Two of the seven major clades identified in the phylogenies based on mitochondrial and combined datasets (Clades 3 and 4) were recovered as monophyletic in ML and BI analyses of RAG1 with moderate to high support (>70BT, 1 pp). Clade 2 was not only recovered as monophyletic in any of these analyses. Clades 1, 5, 6 and 7 also were not monophyletic as some species, for instance, *C*. *elongata*, *C*. *ohridana*, *C*. *zanandreai* (Clade 1), *C*. *calderoni* (Clade 5), *C*. *evreni* (Clade 6) and *Cobitis* sp. Soysallı (Clade 7), were recovered as independent lineages. Unlike in the mtDNA gene tree (see Fig 2), many of the *Cobitis* species resolved as non-monophyletic groups in the RAG1 gene tree. However, some of the highly divergent lineages recovered in the mitochondrial analysis, namely *Cobitis* sp. Aksu-Eğirdir (76BT, 1pp) and *Cobitis* sp. Menderes (99BT, 1pp), were also highly divergent in the nuclear phylogenies, supporting the idea that these lineages are potentially undescribed species.

### Species delimitation and K2P genetic divergence

Although we obtained a wide range of K2P values with cyt b among the seven major clades, mean values between these clades were always greater than 8% (mean 11.5 ± 1.24%, range 8.92–16.20%), much higher than mean values between species, which were always less than 3% (mean 0.59 ± 0.52%, range 0–2.61%). In four of the major clades, we observed higher cyt b distances between certain lineages than between recognized species of *Cobitis* (K2P >2–3%). None of these lineages clustered with any of the *a priori* defined species (Fig 2).

Species delimitation analysis using the bPTP approach, and the phylogeny derived from the complete cyt b dataset, identified at least 49 weakly to strongly supported lineages (0.02–1pp), whereas the more conservative mPTP analysis identified 33 lineages. In many cases, individual lineages detected in the bPTP analysis corresponded to one of the morphologically identified species. In contrast, in the mPTP analysis, many of the *a priori* morphologically defined species grouped together within individual lineages.

Most of the highly differentiated lineages obtained in our study (i.e. *Cobitis* sp. Aksu-Eğirdir, *Cobitis* sp. Ceyhan, *Cobitis* sp. Gölbasi, *Cobitis* sp. Menderes, *Cobitis* sp. Sapanca, and *Cobitis* sp. Soysalli,) had been previously identified by [10] as potential species candidates. Our bPTP analysis strongly supported these lineages with pp values between 0.81 and 1. Other highly differentiated lineages found in our study, namely *Cobitis* sp. Marmara-Black Sea and *Cobitis* sp. Manyas, were not analysed by [10]. These two lineages were moderately to strongly supported with pp values of 0.23 and 0.85, respectively (Fig 2).

We also found some discrepancies between our bPTP results and some of the previously described species. Specifically, some species considered as Central European (*C*. *elongatoides*, *C*. *satunini* and *C*. *tanaitica*), Adriatic (*C*. *ohridana*) or Greek (*C*. *arachthosensis*, *C*. *hellenica*, *C*. *stephanidisi* and *C*. *vardarensis*) were not well supported as monophyletic lineages (<0.36 pp) or grouped together in lineages according to geographic location (Fig 2 and Table 1).

## Discussion

Our mitochondrial and combined results based on the analysis of ~89% of the recognized Western Palearctic *Cobitis* species identified seven highly distinctive clades, Clades 1–7, which, in some cases, were corroborated by the nuclear data. These major clades, and the species comprising them, are largely allopatric and correspond to groups recovered in previous studies with restricted sampling [16], [27], [29]. Our attempt to use molecular data to assess the number of *Cobitis* species in the Western Palearctic provides evidence for some incongruence between *a priori* morphologically defined species and genetically identified lineages. The Western Palearctic loaches exhibit a complex phylogenetic pattern with multiple divergent lineages that not only supports the existence of the six candidate species suggested by [10] on the basis of COI sequences but also two additional candidate species, which were detected due to the extended sampling in Turkey conducted in this study.

### Species delimitation

As described above, we used PTP-based methods to identify putative *Cobitis* species. However, to fully discuss whether identified lineages qualify as strong species candidates, other criteria must also be considered. To do this, we applied criteria successfully used in other freshwater fishes, such as cyprinids or siluriforms [39–40], including i) prior established taxonomy, ii) K2P distances, iii) phylogenetic congruence between mitochondrial and nuclear genes and iv) geographic congruence and range disjunction. Three lineages with high support in the bPTP analysis and moderate to high K2P distances for cyt b (> 2–3%) were highlighted as potentially new taxa: *Cobitis* sp. Gölbasi, *Cobitis* sp. Sapanca, and *Cobitis* sp. Soysallı. However, each of these lineages consist of a single individual. As they have already been identified as candidate species by [10], we will not discuss them further here.

Although we observed a broad range of molecular divergence values, all analyses showed higher mean K2P cyt b divergences among major *Cobitis* clades than within species. Based on the 49 *Cobitis* lineages identified by bPTP, we found a mean K2P value of 0.59% for cyt b intraspecific divergence, similar to the value found for COI divergence (0.82%) [10] (Table 1). In our conservative interpretation, which does not consider lineages comprised of single individuals as new species, Clades 2, 3, 6 and 7 each contain at least one potentially undescribed species: *Cobitis* sp. Manyas, *Cobitis* sp. Marmara-Black Sea, *Cobitis* sp. Menderes, *Cobitis* sp. Ceyhan and *Cobitis* sp. Aksu-Eğirdir. Of these, *Cobitis* sp. Aksu-Eğirdir, *Cobitis* sp. Ceyhan and *Cobitis* sp. Menderes were moderately to well supported in the bPTP analysis, fairly well supported in the mitochondrial and nuclear phylogenies and showed K2P cyt-b distances between 3 and 5%. *Cobitis* sp. Aksu-Eğirdir is restricted to the Aksu river drainage, a current outflow of Lake Eğirdir in Southern Anatolia. *Cobitis* sp. Ceyhan and *Cobitis* sp. Menderes also inhabit particular drainages in restricted areas or lakes and fulfil all criteria to be recognized as new species. *Cobitis* sp. Manyas occurs in sympatry with *C*. *puncticulata*. Although K2P distances revealed the distinctiveness of *Cobitis* sp. Manyas, the nuclear phylogeny did not support it as a distinct lineage. Unlike the first three potentially new *Cobitis* species mentioned above, *Cobitis* sp. Manyas is not as geographically restricted and, therefore, does not satisfy many of the criteria to be considered a new species. Indeed, all available evidence suggests that *Cobitis* sp. Manyas represents a divergent population of *C*. *puncticulata*. Similarly, *Cobitis* sp. Marmara-Black Sea is widely distributed across drainages that terminate in the Marmara and Black seas. In spite of its mitochondrial differentiation, it was only weakly supported in the bPTP analyses and not supported at all in the nuclear phylogenies. Therefore, the *Cobitis* sp. Marmara-Black Sea lineage also does not satisfied all criteria to be considered a candidate new species. Our phylogenetic analyses suggest that this lineage is most closely related to *C*. *taenia*, a Central European species well known to consist of populations that easily hybridize with other *Cobitis* species [41,42]. A study of the phylogeography of *C*. *taenia* populations revealed an old lineage inhabiting drainages of the northern Black Sea that did not contribute to the recolonization of Europe [43]. However, our results highlight a new southern Ponto-Caspian lineage that may provide insights on European *Cobitis* recolonization. The high levels of genetic variation and the complex phylogenetic relationships found in the Turkish *Cobitis* species suggest the need for further phylogeographic evaluation.

In addition to the potentially new species, we also found some discrepancies between our criteria for species delimitation and *a priori* defined species (Table 1). In most cases, morphologically defined species satisfied some of the adopted criteria, with nuclear phylogenetic support being the most commonly violated criterion. Of the previously defined species, *C*.*elongatoides* and *C*. *tanaitica* lacked nuclear support and had low bPTP values (<50%). These species are well known for their tendency to hybridize and have not accumulated large genetic diversity [44–45]. Similarly, *C*. *ohridana* is also considered a potential species of the hybrid complexes formed by *C*.*elongatoides* and *C*. *tanaitica* [30, 46]. In the case of *C*. *vardarensis* and *C*. *stephanidisi*, which recovered as sister species in the mitochondrial phylogeny, none of the other criteria support their delimitation as distinct species. However, in this case, a hybrid complex has not been described for this species. Indeed, *C*. *stephanidisi* has a highly restricted distribution: it is exclusively found in a single karstic spring in Greece (Kefakovriso, in Velestino village) [15]. Given its distribution, this species likely represents an offshoot of the more widely distributed *C*. *vardarensis*. The time elapsed since their split is likely not long enough to show monophyly with the nuclear genes. However, in some cases, such as with the Greek species *C*. *hellenica* and *C*. *arachthosensis*, none of the criteria were achieved (Figs 2–4 and Table 1). Therefore, the independent specific status of *C*. *hellenica* and *C*. *arachthosensis* is unsupported, in agreement with findings reported in previous studies [27, 29]. The pigmentation pattern, especially in young specimens, represents a major difference between the two species [15]. The adjacent distribution of the species and their genetic similarity suggest a recent split and/or recent gene flow leading to unsorted lineages.

### Major *Cobitis* clades in the Western Palearctic

On the basis of patterns of endemism per drainage and observed phylogenetic relationships within Western Palearctic *Cobitis* identified seven major areas of diversification can be identified: Adriatic and upper Danube drainages (Clade 1), Central and Eastern Europe and Asian Marmara-Black-Caspian Sea basin (Clade 2), eastern Greece-western Turkey (Clade 3), western Greece (Clade 4), Iberian-Moroccan drainages (Clade 5), easternmost Mediterranean, Iranian and Euphrates drainages (Clade 6), and Central and Southwest Anatolia (Clade 7) (Figs 2–4 and S1 Fig). Mediterranean peninsulas (Italian and Iberian, Clades 1 and 5) and Central European drainages (Clade 2) hold lower numbers of spined loaches when compared to the Balkan and Eastern Mediterranean drainages. The early isolation of the Iberian and Italian peninsulas and the lack of subsequent connection with the geographically closest ichthyofauna from Central Europe or North Africa most likely promote their high endemicity and low number of species observed in the peninsulas. Our data confirm that the Adriatic region and the Italian and Iberian peninsulas represent long-term persistence areas of spined loaches; however but contrary to what has been observed for other vertebrates [47], these areas do not represent refugia or sources of evolutionary assemblages that colonized Northern Europe at any period. The long-term isolation of the ichthyofauna inhabiting these areas favoured their genetic differentiation and promoted their high level of endemicity. In general, the major *Cobitis* clades, and the species comprising them, are allopatric with the exception of some of the Eastern Mediterranean species inhabiting Turkey and Greece. The observed distribution of species found in Turkish drainages indicate that these habitats are not continuous or linear as the distribution pattern of some current *Cobitis* species appear to correspond to ancient geological units. Drainages occupying geologically active areas, particularly boundary zones between plates such as the river Ceyhan [48], contain different taxa i.e. *C*. *evreni* and *Cobitis* sp. Ceyhan. Furthermore, the observed high endemicity of spined loaches of Clade 7 (7 species and 2 newly proposed species) indicate that the lacustrine system of Central Anatolia as a geologically complex area. Within this are, Lake Beysheir is one the places with the highest number of *Cobitis* species. The phylogenetic pattern found in our study supports the importance of geological history for the speciation of *Cobitis* in the Western Palearctic. Congruent spatial genetic subdivisions have been reported for other vertebrates, including water frogs and lizards, in the Eastern Mediterranean [49–51]. Our results, therefore, provide further evidence of a common pattern of co-distributed taxa within this region.

Overall, our biodiversity assessment indicates that Turkey has the highest level of genetic diversity and endemicity of spined loaches in the Western Palearctic followed by the Balkan region. The remarkable richness observed for this group, which has also been observed for other Eastern Mediterranean groups of freshwater fishes, including cyprinids and aphaniids [13], [52], [53], and has highlighted Turkey as a present-day biodiversity hotspot.

## Supporting information

S1 Fig Phylogenetic tree of the combined dataset (cyt b + RAG1) (N = 215) for the Western Palearctic *Cobitis* species and all species included in the *Cobitis* sensu lato group as defined by Perdices et al. [29] to show their relationships in a broader phylogenetic context. Symbols on branches correspond to bootstrap values (BT): Black dots >95 BT/pp, orange dots 85–95 BT/pp, grey dots <85–50 BT/pp. Major *Cobitis* clades containing Asia Minor species are shown in a grey box.
(EPS)

S1 Table List of new specimens analysed in this study with corresponding collection information. Group refers to major Clades 1–7 obtained in this study.
(XLS)

S2 Table List of specimens obtained from GenBank with corresponding collection information. Group refers to major Clades 1–7 obtained in this study.
(XLS)
